# Supplementary figures and images for: Efficacy of various survey methods to detect an experimental population of spot-tailed earless lizards: A case study
Source: PLoS One. 2025 Nov 17;20(11):e0336129. doi: 10.1371/journal.pone.0336129 (PMC12622777; doi:10.1371/journal.pone.0336129)

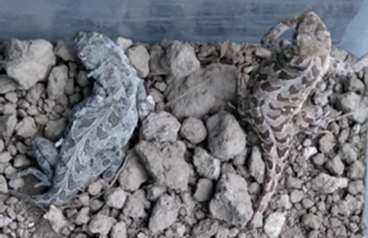

Supplement: S1 Photo — Plateau STEL (right) are caramel colored and Tamaulipan STEL (left) are a slate gray color. (PNG) [file pone.0336129.s001.png]
