# Supplementary material for: Efficacy of various survey methods to detect an experimental population of spot-tailed earless lizards: A case study
Source: PLoS One. 2025 Nov 17;20(11):e0336129. doi: 10.1371/journal.pone.0336129 (PMC12622777; doi:10.1371/journal.pone.0336129)
Supplement: Supplementary material 1 — Code for statistical analysis of hypothesis 1: testing variation detection probability of lizards across known densities using two different capture methods: road cruising and visual searches. (DOCX) [file pone.0336129.s002.docx]

**Supplementary material**

**Supplementary material 1.** Code for statistical analysis of hypothesis 1: testing variation detection probability of lizards across known densities using two different capture methods: road cruising and visual searches.

**Supplementary Material**

# Code for statistical analysis of hypothesis 1: testing variation detection probability of lizards across known densities using two different capture methods: road cruising and visual searches

 Code

Paper: Efficacy of various survey methods to detect an experimental population of spot-tailed earless lizards: A case study

## Packages

Code

## Introduction

In this analysis, we estimate the probability of detecting lizards across known densities using two different capture methods: **road cruising** and **visual searches**. The study uses data from a controlled field experiment in which known numbers of lizards were released into enclosed plots.

We fit a binomial model with detection counts as the response and compare a model with nested random effects against a simpler fixed-effects model to evaluate detection differences between methods.

# 1. Data preparation

We start by loading the dataset and preparing the data for modeling.

Code

Now, we filter the data to include only the capture methods of interest (TRT 7 = Systematic visual searches and TRT 8 = Road Cruising), remove missing values, and summarize lizard detections per plot.

Code

tibble [45 × 5] (S3: tbl_df/tbl/data.frame)

$ DENSITY : chr [1:45] "10" "10" "10" "10" ...

$ TRT : chr [1:45] "7" "7" "7" "7" ...

$ PLOT : chr [1:45] "1" "2" "3" "4" ...

$ Detections: num [1:45] 0 0 0 0 0 0 0 1 0 0 ...

$ Days : num [1:45] 3 3 3 3 3 3 3 3 3 3 ...

# 2. Model fitting

We fit two binomial models using the brms package:

- One with **nested random effects** (plot nested within treatment),
- Another with only **fixed effects** for DENSITY and TRT.

## 2.1 Model 1: Nested Random effects

Code

Running "C:/PROGRA~1/R/R-45~1.1/bin/x64/Rcmd.exe" SHLIB foo.c

using C compiler: 'gcc.exe (GCC) 14.2.0'

gcc -I"C:/PROGRA~1/R/R-45~1.1/include" -DNDEBUG -I"C:/Users/andradegp/AppData/Local/R/win-library/4.5/Rcpp/include/" -I"C:/Users/andradegp/AppData/Local/R/win-library/4.5/RcppEigen/include/" -I"C:/Users/andradegp/AppData/Local/R/win-library/4.5/RcppEigen/include/unsupported" -I"C:/Users/andradegp/AppData/Local/R/win-library/4.5/BH/include" -I"C:/Users/andradegp/AppData/Local/R/win-library/4.5/StanHeaders/include/src/" -I"C:/Users/andradegp/AppData/Local/R/win-library/4.5/StanHeaders/include/" -I"C:/Users/andradegp/AppData/Local/R/win-library/4.5/RcppParallel/include/" -DRCPP_PARALLEL_USE_TBB=1 -I"C:/Users/andradegp/AppData/Local/R/win-library/4.5/rstan/include" -DEIGEN_NO_DEBUG -DBOOST_DISABLE_ASSERTS -DBOOST_PENDING_INTEGER_LOG2_HPP -DSTAN_THREADS -DUSE_STANC3 -DSTRICT_R_HEADERS -DBOOST_PHOENIX_NO_VARIADIC_EXPRESSION -D_HAS_AUTO_PTR_ETC=0 -include "C:/Users/andradegp/AppData/Local/R/win-library/4.5/StanHeaders/include/stan/math/prim/fun/Eigen.hpp" -std=c++1y -I"C:/rtools45/x86_64-w64-mingw32.static.posix/include" -O2 -Wall -std=gnu2x -mfpmath=sse -msse2 -mstackrealign -c foo.c -o foo.o

cc1.exe: warning: command-line option '-std=c++14' is valid for C++/ObjC++ but not for C

In file included from C:/Users/andradegp/AppData/Local/R/win-library/4.5/RcppEigen/include/Eigen/Core:19,

from C:/Users/andradegp/AppData/Local/R/win-library/4.5/RcppEigen/include/Eigen/Dense:1,

from C:/Users/andradegp/AppData/Local/R/win-library/4.5/StanHeaders/include/stan/math/prim/fun/Eigen.hpp:22,

from <command-line>:

C:/Users/andradegp/AppData/Local/R/win-library/4.5/RcppEigen/include/Eigen/src/Core/util/Macros.h:679:10: fatal error: cmath: No such file or directory

679 | #include <cmath>

| ^~~~~~~

compilation terminated.

make: *** [C:/PROGRA~1/R/R-45~1.1/etc/x64/Makeconf:289: foo.o] Error 1

SAMPLING FOR MODEL 'anon_model' NOW (CHAIN 1).

Chain 1:

Chain 1: Gradient evaluation took 3.6e-05 seconds

Chain 1: 1000 transitions using 10 leapfrog steps per transition would take 0.36 seconds.

Chain 1: Adjust your expectations accordingly!

Chain 1:

Chain 1:

Chain 1: Iteration: 1 / 6000 [ 0%] (Warmup)

Chain 1: Iteration: 600 / 6000 [ 10%] (Warmup)

Chain 1: Iteration: 1001 / 6000 [ 16%] (Sampling)

Chain 1: Iteration: 1600 / 6000 [ 26%] (Sampling)

Chain 1: Iteration: 2200 / 6000 [ 36%] (Sampling)

Chain 1: Iteration: 2800 / 6000 [ 46%] (Sampling)

Chain 1: Iteration: 3400 / 6000 [ 56%] (Sampling)

Chain 1: Iteration: 4000 / 6000 [ 66%] (Sampling)

Chain 1: Iteration: 4600 / 6000 [ 76%] (Sampling)

Chain 1: Iteration: 5200 / 6000 [ 86%] (Sampling)

Chain 1: Iteration: 5800 / 6000 [ 96%] (Sampling)

Chain 1: Iteration: 6000 / 6000 [100%] (Sampling)

Chain 1:

Chain 1: Elapsed Time: 0.838 seconds (Warm-up)

Chain 1: 3.698 seconds (Sampling)

Chain 1: 4.536 seconds (Total)

Chain 1:

SAMPLING FOR MODEL 'anon_model' NOW (CHAIN 2).

Chain 2:

Chain 2: Gradient evaluation took 1.9e-05 seconds

Chain 2: 1000 transitions using 10 leapfrog steps per transition would take 0.19 seconds.

Chain 2: Adjust your expectations accordingly!

Chain 2:

Chain 2:

Chain 2: Iteration: 1 / 6000 [ 0%] (Warmup)

Chain 2: Iteration: 600 / 6000 [ 10%] (Warmup)

Chain 2: Iteration: 1001 / 6000 [ 16%] (Sampling)

Chain 2: Iteration: 1600 / 6000 [ 26%] (Sampling)

Chain 2: Iteration: 2200 / 6000 [ 36%] (Sampling)

Chain 2: Iteration: 2800 / 6000 [ 46%] (Sampling)

Chain 2: Iteration: 3400 / 6000 [ 56%] (Sampling)

Chain 2: Iteration: 4000 / 6000 [ 66%] (Sampling)

Chain 2: Iteration: 4600 / 6000 [ 76%] (Sampling)

Chain 2: Iteration: 5200 / 6000 [ 86%] (Sampling)

Chain 2: Iteration: 5800 / 6000 [ 96%] (Sampling)

Chain 2: Iteration: 6000 / 6000 [100%] (Sampling)

Chain 2:

Chain 2: Elapsed Time: 0.919 seconds (Warm-up)

Chain 2: 2.251 seconds (Sampling)

Chain 2: 3.17 seconds (Total)

Chain 2:

SAMPLING FOR MODEL 'anon_model' NOW (CHAIN 3).

Chain 3:

Chain 3: Gradient evaluation took 4.9e-05 seconds

Chain 3: 1000 transitions using 10 leapfrog steps per transition would take 0.49 seconds.

Chain 3: Adjust your expectations accordingly!

Chain 3:

Chain 3:

Chain 3: Iteration: 1 / 6000 [ 0%] (Warmup)

Chain 3: Iteration: 600 / 6000 [ 10%] (Warmup)

Chain 3: Iteration: 1001 / 6000 [ 16%] (Sampling)

Chain 3: Iteration: 1600 / 6000 [ 26%] (Sampling)

Chain 3: Iteration: 2200 / 6000 [ 36%] (Sampling)

Chain 3: Iteration: 2800 / 6000 [ 46%] (Sampling)

Chain 3: Iteration: 3400 / 6000 [ 56%] (Sampling)

Chain 3: Iteration: 4000 / 6000 [ 66%] (Sampling)

Chain 3: Iteration: 4600 / 6000 [ 76%] (Sampling)

Chain 3: Iteration: 5200 / 6000 [ 86%] (Sampling)

Chain 3: Iteration: 5800 / 6000 [ 96%] (Sampling)

Chain 3: Iteration: 6000 / 6000 [100%] (Sampling)

Chain 3:

Chain 3: Elapsed Time: 0.743 seconds (Warm-up)

Chain 3: 4.363 seconds (Sampling)

Chain 3: 5.106 seconds (Total)

Chain 3:

SAMPLING FOR MODEL 'anon_model' NOW (CHAIN 4).

Chain 4:

Chain 4: Gradient evaluation took 2.1e-05 seconds

Chain 4: 1000 transitions using 10 leapfrog steps per transition would take 0.21 seconds.

Chain 4: Adjust your expectations accordingly!

Chain 4:

Chain 4:

Chain 4: Iteration: 1 / 6000 [ 0%] (Warmup)

Chain 4: Iteration: 600 / 6000 [ 10%] (Warmup)

Chain 4: Iteration: 1001 / 6000 [ 16%] (Sampling)

Chain 4: Iteration: 1600 / 6000 [ 26%] (Sampling)

Chain 4: Iteration: 2200 / 6000 [ 36%] (Sampling)

Chain 4: Iteration: 2800 / 6000 [ 46%] (Sampling)

Chain 4: Iteration: 3400 / 6000 [ 56%] (Sampling)

Chain 4: Iteration: 4000 / 6000 [ 66%] (Sampling)

Chain 4: Iteration: 4600 / 6000 [ 76%] (Sampling)

Chain 4: Iteration: 5200 / 6000 [ 86%] (Sampling)

Chain 4: Iteration: 5800 / 6000 [ 96%] (Sampling)

Chain 4: Iteration: 6000 / 6000 [100%] (Sampling)

Chain 4:

Chain 4: Elapsed Time: 0.732 seconds (Warm-up)

Chain 4: 5.98 seconds (Sampling)

Chain 4: 6.712 seconds (Total)

Chain 4:

Code

Family: binomial

Links: mu = logit

Formula: Detections | trials(Days) ~ DENSITY * TRT + (1 | TRT/PLOT)

Data: mod_data (Number of observations: 45)

Draws: 4 chains, each with iter = 6000; warmup = 1000; thin = 10;

total post-warmup draws = 2000

Multilevel Hyperparameters:

~TRT (Number of levels: 2)

Estimate Est.Error l-90% CI u-90% CI Rhat Bulk_ESS Tail_ESS

sd(Intercept) 0.89 0.84 0.04 2.55 1.00 1757 1971

~TRT:PLOT (Number of levels: 9)

Estimate Est.Error l-90% CI u-90% CI Rhat Bulk_ESS Tail_ESS

sd(Intercept) 0.67 0.50 0.06 1.61 1.00 1930 1891

Regression Coefficients:

Estimate Est.Error l-90% CI u-90% CI Rhat Bulk_ESS Tail_ESS

Intercept -4.62 1.51 -6.98 -2.19 1.00 2023 2010

DENSITY20 -0.33 1.58 -3.02 2.12 1.00 2074 1948

DENSITY30 -0.14 1.54 -2.76 2.26 1.00 1869 1973

DENSITY40 3.07 1.22 1.09 5.13 1.00 1963 1867

DENSITY5 -0.67 1.64 -3.43 1.98 1.00 2014 1930

TRT8 1.72 1.59 -0.99 4.22 1.00 2288 2013

DENSITY20:TRT8 1.00 1.69 -1.72 3.77 1.00 2103 1919

DENSITY30:TRT8 1.45 1.59 -1.06 4.14 1.00 1823 2035

DENSITY40:TRT8 -0.66 1.33 -2.80 1.49 1.00 1946 1715

DENSITY5:TRT8 0.37 1.74 -2.39 3.28 1.00 2001 1759

Draws were sampled using sampling(NUTS). For each parameter, Bulk_ESS

and Tail_ESS are effective sample size measures, and Rhat is the potential

scale reduction factor on split chains (at convergence, Rhat = 1).

## 2.2 Model 2: Fixed effects only

Code

Running "C:/PROGRA~1/R/R-45~1.1/bin/x64/Rcmd.exe" SHLIB foo.c

using C compiler: 'gcc.exe (GCC) 14.2.0'

gcc -I"C:/PROGRA~1/R/R-45~1.1/include" -DNDEBUG -I"C:/Users/andradegp/AppData/Local/R/win-library/4.5/Rcpp/include/" -I"C:/Users/andradegp/AppData/Local/R/win-library/4.5/RcppEigen/include/" -I"C:/Users/andradegp/AppData/Local/R/win-library/4.5/RcppEigen/include/unsupported" -I"C:/Users/andradegp/AppData/Local/R/win-library/4.5/BH/include" -I"C:/Users/andradegp/AppData/Local/R/win-library/4.5/StanHeaders/include/src/" -I"C:/Users/andradegp/AppData/Local/R/win-library/4.5/StanHeaders/include/" -I"C:/Users/andradegp/AppData/Local/R/win-library/4.5/RcppParallel/include/" -DRCPP_PARALLEL_USE_TBB=1 -I"C:/Users/andradegp/AppData/Local/R/win-library/4.5/rstan/include" -DEIGEN_NO_DEBUG -DBOOST_DISABLE_ASSERTS -DBOOST_PENDING_INTEGER_LOG2_HPP -DSTAN_THREADS -DUSE_STANC3 -DSTRICT_R_HEADERS -DBOOST_PHOENIX_NO_VARIADIC_EXPRESSION -D_HAS_AUTO_PTR_ETC=0 -include "C:/Users/andradegp/AppData/Local/R/win-library/4.5/StanHeaders/include/stan/math/prim/fun/Eigen.hpp" -std=c++1y -I"C:/rtools45/x86_64-w64-mingw32.static.posix/include" -O2 -Wall -std=gnu2x -mfpmath=sse -msse2 -mstackrealign -c foo.c -o foo.o

cc1.exe: warning: command-line option '-std=c++14' is valid for C++/ObjC++ but not for C

In file included from C:/Users/andradegp/AppData/Local/R/win-library/4.5/RcppEigen/include/Eigen/Core:19,

from C:/Users/andradegp/AppData/Local/R/win-library/4.5/RcppEigen/include/Eigen/Dense:1,

from C:/Users/andradegp/AppData/Local/R/win-library/4.5/StanHeaders/include/stan/math/prim/fun/Eigen.hpp:22,

from <command-line>:

C:/Users/andradegp/AppData/Local/R/win-library/4.5/RcppEigen/include/Eigen/src/Core/util/Macros.h:679:10: fatal error: cmath: No such file or directory

679 | #include <cmath>

| ^~~~~~~

compilation terminated.

make: *** [C:/PROGRA~1/R/R-45~1.1/etc/x64/Makeconf:289: foo.o] Error 1

SAMPLING FOR MODEL 'anon_model' NOW (CHAIN 1).

Chain 1:

Chain 1: Gradient evaluation took 2.6e-05 seconds

Chain 1: 1000 transitions using 10 leapfrog steps per transition would take 0.26 seconds.

Chain 1: Adjust your expectations accordingly!

Chain 1:

Chain 1:

Chain 1: Iteration: 1 / 6000 [ 0%] (Warmup)

Chain 1: Iteration: 600 / 6000 [ 10%] (Warmup)

Chain 1: Iteration: 1001 / 6000 [ 16%] (Sampling)

Chain 1: Iteration: 1600 / 6000 [ 26%] (Sampling)

Chain 1: Iteration: 2200 / 6000 [ 36%] (Sampling)

Chain 1: Iteration: 2800 / 6000 [ 46%] (Sampling)

Chain 1: Iteration: 3400 / 6000 [ 56%] (Sampling)

Chain 1: Iteration: 4000 / 6000 [ 66%] (Sampling)

Chain 1: Iteration: 4600 / 6000 [ 76%] (Sampling)

Chain 1: Iteration: 5200 / 6000 [ 86%] (Sampling)

Chain 1: Iteration: 5800 / 6000 [ 96%] (Sampling)

Chain 1: Iteration: 6000 / 6000 [100%] (Sampling)

Chain 1:

Chain 1: Elapsed Time: 0.241 seconds (Warm-up)

Chain 1: 1.152 seconds (Sampling)

Chain 1: 1.393 seconds (Total)

Chain 1:

SAMPLING FOR MODEL 'anon_model' NOW (CHAIN 2).

Chain 2:

Chain 2: Gradient evaluation took 1.4e-05 seconds

Chain 2: 1000 transitions using 10 leapfrog steps per transition would take 0.14 seconds.

Chain 2: Adjust your expectations accordingly!

Chain 2:

Chain 2:

Chain 2: Iteration: 1 / 6000 [ 0%] (Warmup)

Chain 2: Iteration: 600 / 6000 [ 10%] (Warmup)

Chain 2: Iteration: 1001 / 6000 [ 16%] (Sampling)

Chain 2: Iteration: 1600 / 6000 [ 26%] (Sampling)

Chain 2: Iteration: 2200 / 6000 [ 36%] (Sampling)

Chain 2: Iteration: 2800 / 6000 [ 46%] (Sampling)

Chain 2: Iteration: 3400 / 6000 [ 56%] (Sampling)

Chain 2: Iteration: 4000 / 6000 [ 66%] (Sampling)

Chain 2: Iteration: 4600 / 6000 [ 76%] (Sampling)

Chain 2: Iteration: 5200 / 6000 [ 86%] (Sampling)

Chain 2: Iteration: 5800 / 6000 [ 96%] (Sampling)

Chain 2: Iteration: 6000 / 6000 [100%] (Sampling)

Chain 2:

Chain 2: Elapsed Time: 0.262 seconds (Warm-up)

Chain 2: 1.06 seconds (Sampling)

Chain 2: 1.322 seconds (Total)

Chain 2:

SAMPLING FOR MODEL 'anon_model' NOW (CHAIN 3).

Chain 3:

Chain 3: Gradient evaluation took 1.2e-05 seconds

Chain 3: 1000 transitions using 10 leapfrog steps per transition would take 0.12 seconds.

Chain 3: Adjust your expectations accordingly!

Chain 3:

Chain 3:

Chain 3: Iteration: 1 / 6000 [ 0%] (Warmup)

Chain 3: Iteration: 600 / 6000 [ 10%] (Warmup)

Chain 3: Iteration: 1001 / 6000 [ 16%] (Sampling)

Chain 3: Iteration: 1600 / 6000 [ 26%] (Sampling)

Chain 3: Iteration: 2200 / 6000 [ 36%] (Sampling)

Chain 3: Iteration: 2800 / 6000 [ 46%] (Sampling)

Chain 3: Iteration: 3400 / 6000 [ 56%] (Sampling)

Chain 3: Iteration: 4000 / 6000 [ 66%] (Sampling)

Chain 3: Iteration: 4600 / 6000 [ 76%] (Sampling)

Chain 3: Iteration: 5200 / 6000 [ 86%] (Sampling)

Chain 3: Iteration: 5800 / 6000 [ 96%] (Sampling)

Chain 3: Iteration: 6000 / 6000 [100%] (Sampling)

Chain 3:

Chain 3: Elapsed Time: 0.284 seconds (Warm-up)

Chain 3: 1.359 seconds (Sampling)

Chain 3: 1.643 seconds (Total)

Chain 3:

SAMPLING FOR MODEL 'anon_model' NOW (CHAIN 4).

Chain 4:

Chain 4: Gradient evaluation took 1.3e-05 seconds

Chain 4: 1000 transitions using 10 leapfrog steps per transition would take 0.13 seconds.

Chain 4: Adjust your expectations accordingly!

Chain 4:

Chain 4:

Chain 4: Iteration: 1 / 6000 [ 0%] (Warmup)

Chain 4: Iteration: 600 / 6000 [ 10%] (Warmup)

Chain 4: Iteration: 1001 / 6000 [ 16%] (Sampling)

Chain 4: Iteration: 1600 / 6000 [ 26%] (Sampling)

Chain 4: Iteration: 2200 / 6000 [ 36%] (Sampling)

Chain 4: Iteration: 2800 / 6000 [ 46%] (Sampling)

Chain 4: Iteration: 3400 / 6000 [ 56%] (Sampling)

Chain 4: Iteration: 4000 / 6000 [ 66%] (Sampling)

Chain 4: Iteration: 4600 / 6000 [ 76%] (Sampling)

Chain 4: Iteration: 5200 / 6000 [ 86%] (Sampling)

Chain 4: Iteration: 5800 / 6000 [ 96%] (Sampling)

Chain 4: Iteration: 6000 / 6000 [100%] (Sampling)

Chain 4:

Chain 4: Elapsed Time: 0.303 seconds (Warm-up)

Chain 4: 1.311 seconds (Sampling)

Chain 4: 1.614 seconds (Total)

Chain 4:

Code

Family: binomial

Links: mu = logit

Formula: Detections | trials(Days) ~ DENSITY * TRT

Data: mod_data (Number of observations: 45)

Draws: 4 chains, each with iter = 6000; warmup = 1000; thin = 10;

total post-warmup draws = 2000

Regression Coefficients:

Estimate Est.Error l-90% CI u-90% CI Rhat Bulk_ESS Tail_ESS

Intercept -4.72 1.14 -6.65 -3.00 1.00 1825 2032

DENSITY20 -0.33 1.54 -2.99 2.03 1.00 1911 1930

DENSITY30 -0.17 1.52 -2.68 2.28 1.00 2030 1649

DENSITY40 2.92 1.19 1.02 4.92 1.00 1785 1845

DENSITY5 -0.67 1.63 -3.49 1.94 1.00 2044 1931

TRT8 2.06 1.14 0.18 3.91 1.00 1911 1859

DENSITY20:TRT8 1.09 1.65 -1.69 3.75 1.00 1962 1854

DENSITY30:TRT8 1.49 1.57 -0.97 4.19 1.00 2110 1972

DENSITY40:TRT8 -0.58 1.28 -2.70 1.52 1.00 1928 1963

DENSITY5:TRT8 0.41 1.76 -2.47 3.33 1.00 2138 1893

Draws were sampled using sampling(NUTS). For each parameter, Bulk_ESS

and Tail_ESS are effective sample size measures, and Rhat is the potential

scale reduction factor on split chains (at convergence, Rhat = 1).

# 3. Model comparison

We use the **Leave-One-Out Cross-Validation (LOO)** to compare model performance. Both models fit similarly well.

Code

elpd_diff se_diff

mod_brm2 0.0 0.0

mod_brm1 -0.3 1.3

Given that both models perform similarly and due to limited data per random effect group, we proceed with the **fixed-effects model**.

## 3.1 Model convergence and fit diagnostics

Code


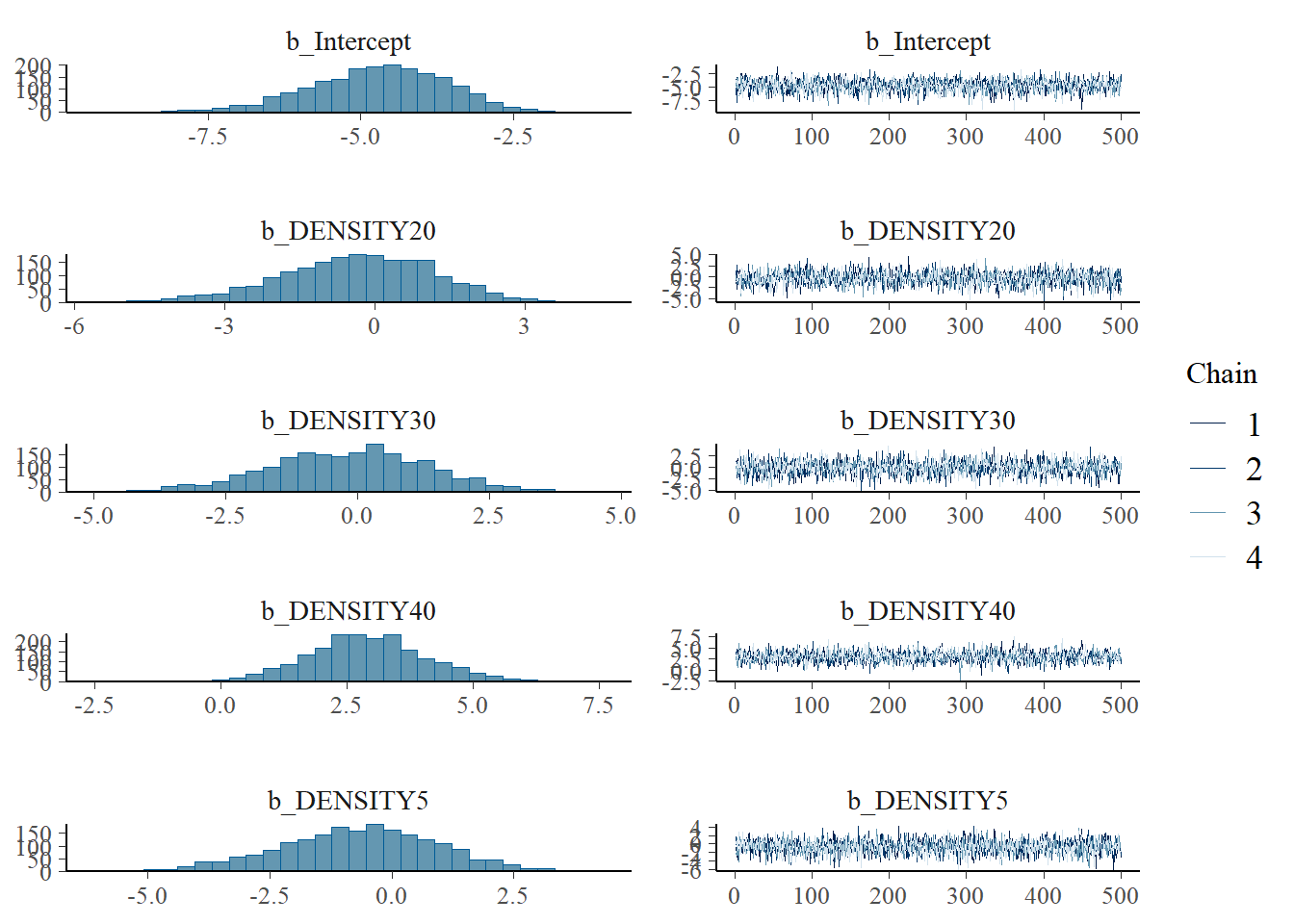

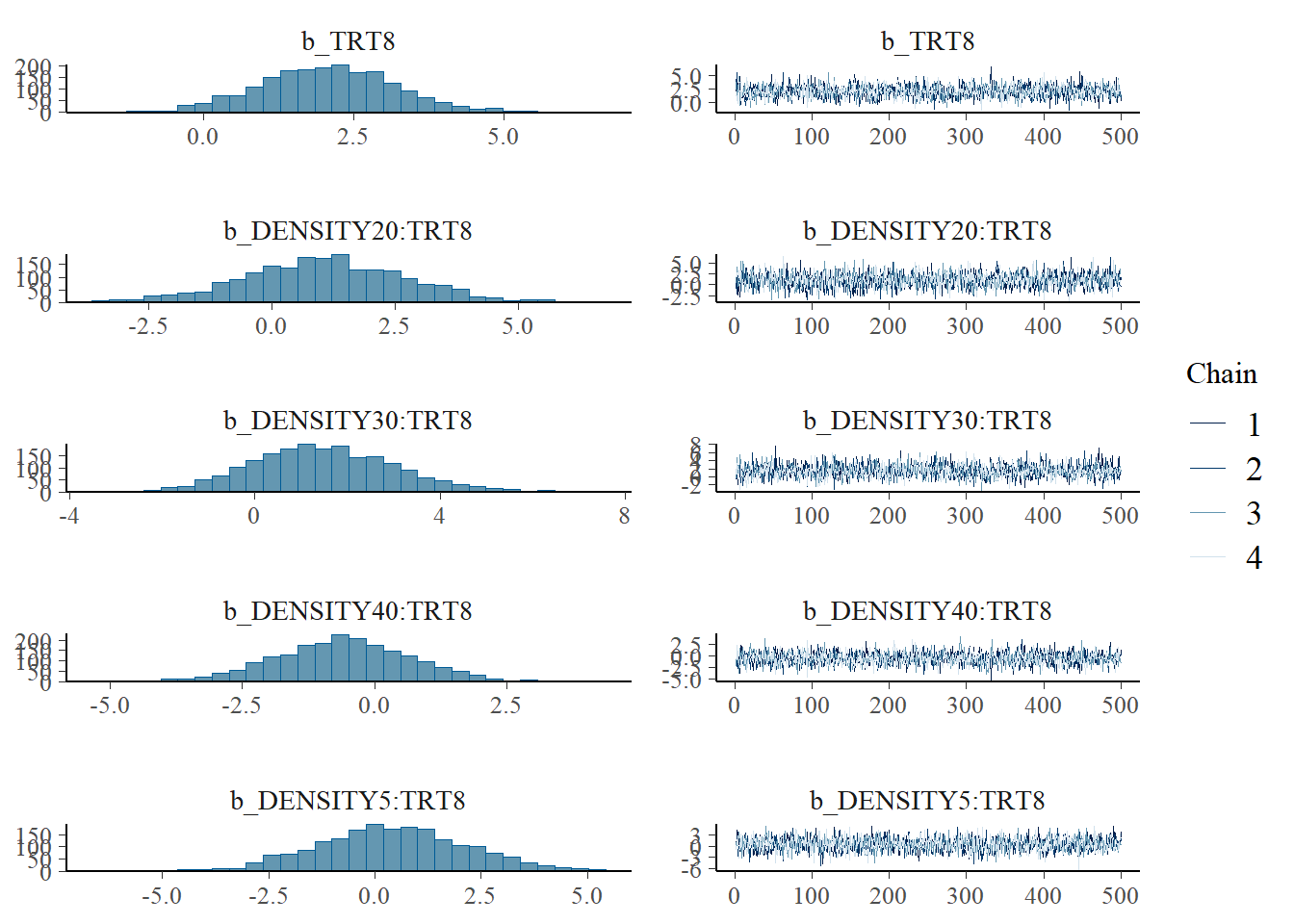
We check model fit using dh_check_brms from the performance package. The argument integer = TRUE is required for binomial responses.

Code


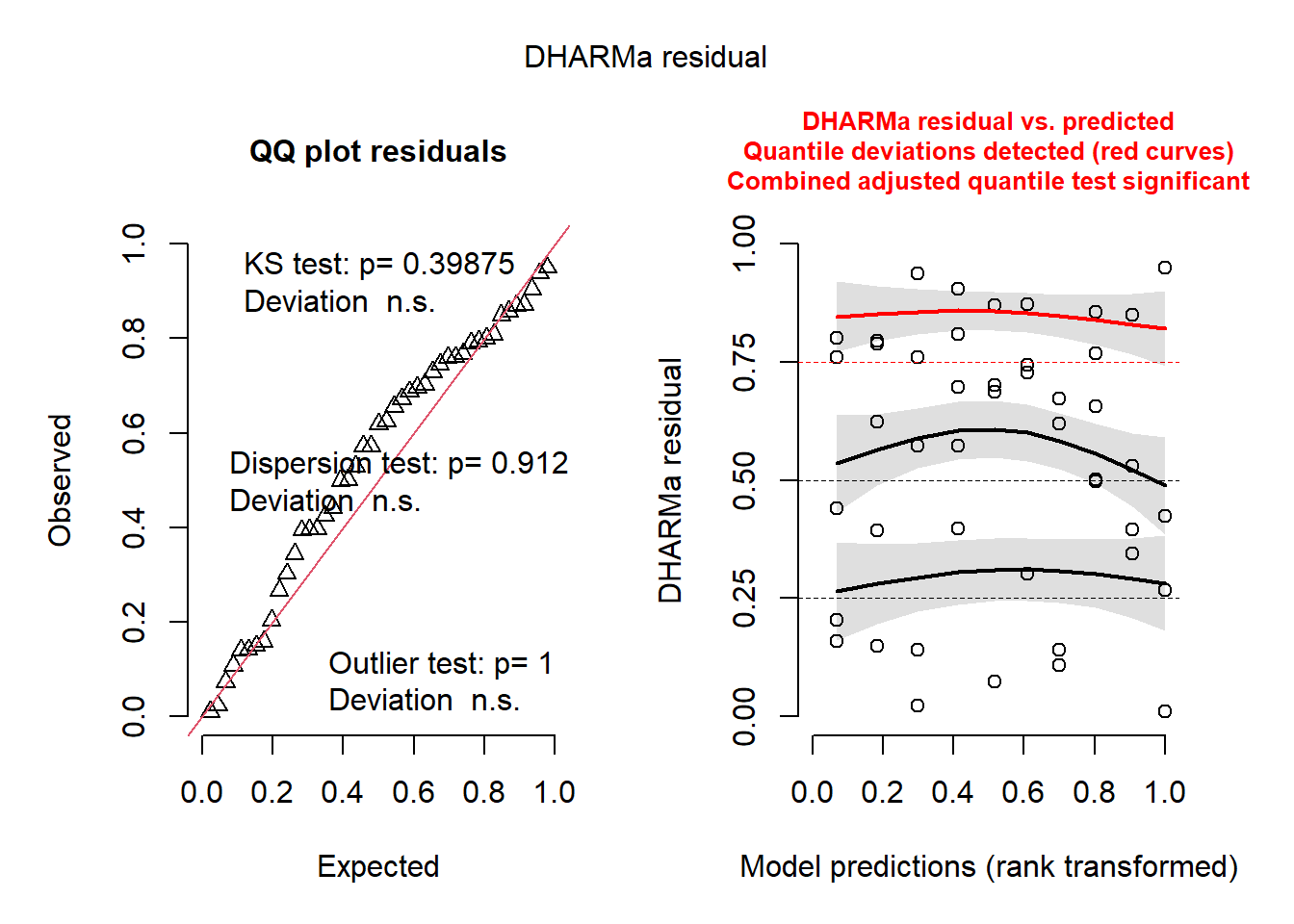
Although the residuals plot of DHARMa vs predicted values shows a deviation, the tests show that it is not significant KS test: p 0.39. Additionally, the trend following the graph does not show to be so problematic that we consider that the model is still valid.

# 4. Model Predictions

We estimate the marginal means (on the response scale) of detection probability by DENSITY within each TRT method and conduct pairwise comparisons. The table presents posterior estimates of odds ratios comparing detection probabilities between animal density levels (e.g., DENSITY10 vs. DENSITY20) for each sampling method: Visual Systematic Search and Road Cruising. Values above 1 indicate greater odds of detection in the first density level of the contrast. The 95% credible intervals (CI) represent the uncertainty around the estimate.

Code

TRT = 7:

DENSITY prob lower.HPD upper.HPD

10 0.00945 2.30e-04 0.0359

20 0.00751 9.80e-06 0.0428

30 0.00850 2.42e-05 0.0479

40 0.14637 3.76e-02 0.2994

5 0.00503 2.99e-06 0.0363

TRT = 8:

DENSITY prob lower.HPD upper.HPD

10 0.06981 2.59e-03 0.1789

20 0.13802 1.31e-02 0.2907

30 0.21097 5.59e-02 0.4195

40 0.42196 1.91e-01 0.6346

5 0.05892 4.21e-04 0.1759

Point estimate displayed: median

Results are back-transformed from the logit scale

HPD interval probability: 0.9

Code

| Pairwise Comparison of Detection Methods | | | | |
| --- | --- | --- | --- | --- |
| Contrast | Reference Method | Odds Ratio | Lower 95% CI | Upper 95% CI |
| DENSITY10 / DENSITY20 | Visual Systematic Search | 1.32 | 0.01 | 9.98 |
| DENSITY10 / DENSITY30 | Visual Systematic Search | 1.13 | 0.01 | 8.51 |
| DENSITY10 / DENSITY40 | Visual Systematic Search | 0.06 | 0.00 | 0.24 |
| DENSITY10 / DENSITY5 | Visual Systematic Search | 1.84 | 0.02 | 16.36 |
| DENSITY20 / DENSITY30 | Visual Systematic Search | 0.88 | 0.00 | 11.44 |
| DENSITY20 / DENSITY40 | Visual Systematic Search | 0.05 | 0.00 | 0.30 |
| DENSITY20 / DENSITY5 | Visual Systematic Search | 1.44 | 0.00 | 20.77 |
| DENSITY30 / DENSITY40 | Visual Systematic Search | 0.05 | 0.00 | 0.34 |
| DENSITY30 / DENSITY5 | Visual Systematic Search | 1.61 | 0.00 | 22.72 |
| DENSITY40 / DENSITY5 | Visual Systematic Search | 33.72 | 0.25 | 342.21 |
| DENSITY10 / DENSITY20 | Road Cruising | 0.48 | 0.01 | 2.14 |
| DENSITY10 / DENSITY30 | Road Cruising | 0.28 | 0.01 | 1.09 |
| DENSITY10 / DENSITY40 | Road Cruising | 0.10 | 0.00 | 0.38 |
| DENSITY10 / DENSITY5 | Road Cruising | 1.22 | 0.01 | 8.19 |
| DENSITY20 / DENSITY30 | Road Cruising | 0.57 | 0.02 | 2.20 |
| DENSITY20 / DENSITY40 | Road Cruising | 0.21 | 0.01 | 0.75 |
| DENSITY20 / DENSITY5 | Road Cruising | 2.51 | 0.06 | 18.70 |
| DENSITY30 / DENSITY40 | Road Cruising | 0.37 | 0.03 | 1.25 |
| DENSITY30 / DENSITY5 | Road Cruising | 4.42 | 0.09 | 29.38 |
| DENSITY40 / DENSITY5 | Road Cruising | 11.64 | 0.41 | 80.47 |

We then convert the results into a data frame for plotting and relabel the methods.

Code

# 5. Model visualization and summary table

The final plot shows the estimated detection probability for each density and method, with 95% credible intervals.

Code


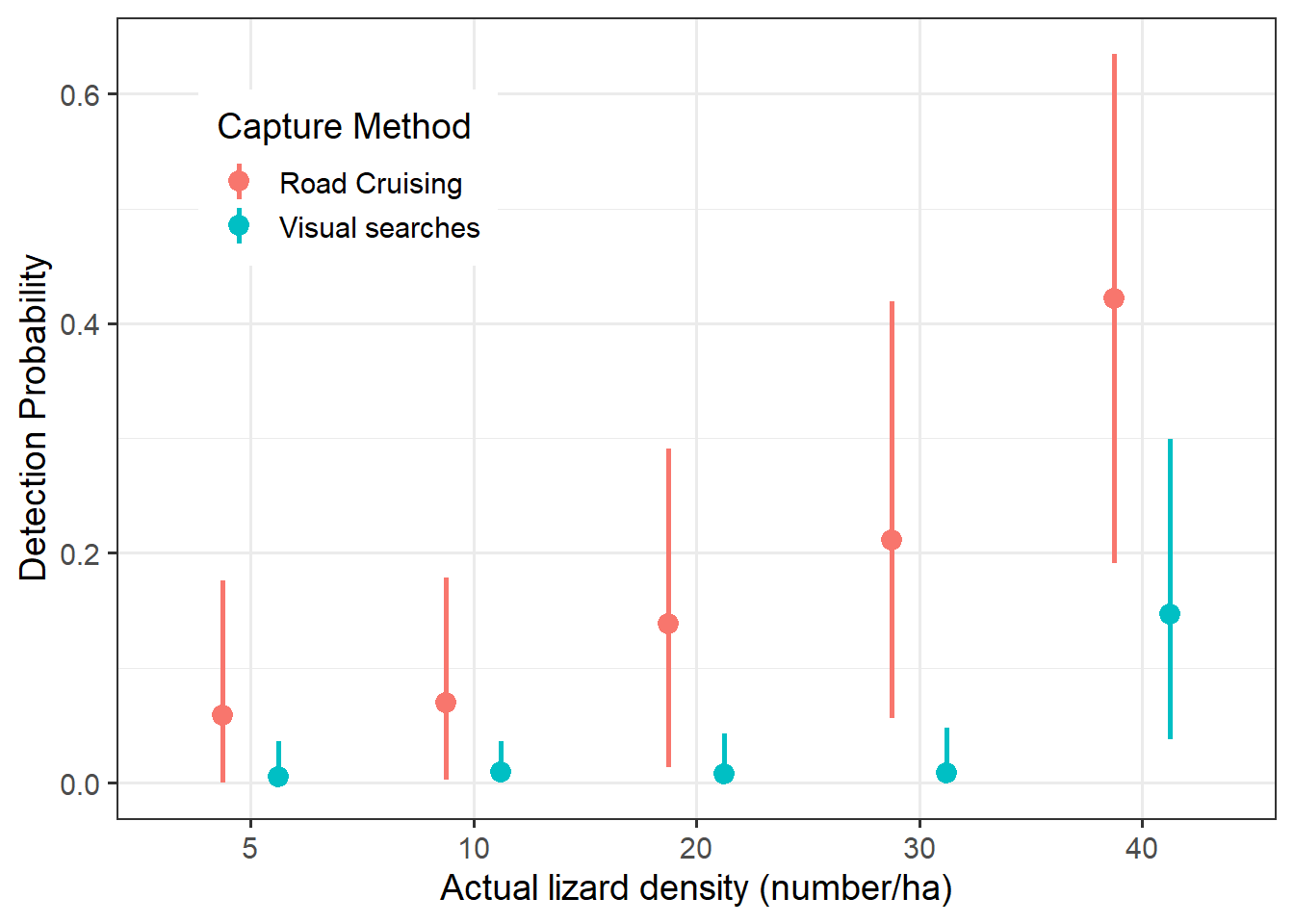
Code

Final model coefficient table

Code

| Coefficient | Estimate | SE | Lower 90% CI | Upper 90% CI | rhat | ess |
| --- | --- | --- | --- | --- | --- | --- |
| (Intercept) | -4.72 | 1.14 | -7.10 | -2.72 | 1.000 | 1828.56 |
| DENSITY20 | -0.33 | 1.54 | -3.57 | 2.50 | 1.002 | 1919.03 |
| DENSITY30 | -0.17 | 1.52 | -3.29 | 2.77 | 0.999 | 2032.66 |
| DENSITY40 | 2.92 | 1.19 | 0.70 | 5.32 | 1.003 | 1781.82 |
| DENSITY5 | -0.67 | 1.63 | -3.93 | 2.41 | 1.000 | 2045.57 |
| TRT8 | 2.06 | 1.14 | -0.15 | 4.37 | 1.000 | 1918.26 |
| DENSITY20:TRT8 | 1.09 | 1.65 | -2.23 | 4.25 | 1.001 | 1964.53 |
| DENSITY30:TRT8 | 1.49 | 1.57 | -1.39 | 4.75 | 1.000 | 2095.81 |
| DENSITY40:TRT8 | -0.58 | 1.28 | -3.06 | 1.90 | 1.000 | 1923.45 |
| DENSITY5:TRT8 | 0.41 | 1.76 | -2.92 | 3.84 | 1.000 | 2140.47 |

# Conclusion

Our analysis indicates that detection probabilities increase with density and vary across capture methods. While both models fit similarly, the simpler fixed-effects model was selected based on practical considerations and effective fit diagnostics.
